# A New Measure of Centrality for Brain Networks

**DOI:** 10.1371/journal.pone.0012200

**Published:** 2010-08-16

**Authors:** Karen E. Joyce, Paul J. Laurienti, Jonathan H. Burdette, Satoru Hayasaka

**Affiliations:** 1 School of Biomedical Engineering and Sciences, Wake Forest University School of Medicine, Winston-Salem, North Carolina, United States of America; 2 Department of Radiology, Wake Forest University School of Medicine, Winston-Salem, North Carolina, United States of America; 3 Department of Biostatistical Sciences, Wake Forest University School of Medicine, Winston-Salem, North Carolina, United States of America; Indiana University, United States of America

## Abstract

Recent developments in network theory have allowed for the study of the structure and function of the human brain in terms of a network of interconnected components. Among the many nodes that form a network, some play a crucial role and are said to be central within the network structure. Central nodes may be identified via centrality metrics, with degree, betweenness, and eigenvector centrality being three of the most popular measures. Degree identifies the most connected nodes, whereas betweenness centrality identifies those located on the most traveled paths. Eigenvector centrality considers nodes connected to other high degree nodes as highly central. In the work presented here, we propose a new centrality metric called leverage centrality that considers the extent of connectivity of a node *relative to the connectivity of its neighbors*. The leverage centrality of a node in a network is determined by the extent to which its immediate neighbors rely on that node for information. Although similar in concept, there are essential differences between eigenvector and leverage centrality that are discussed in this manuscript. Degree, betweenness, eigenvector, and leverage centrality were compared using functional brain networks generated from healthy volunteers. Functional cartography was also used to identify neighborhood hubs (nodes with high degree within a network neighborhood). Provincial hubs provide structure within the local community, and connector hubs mediate connections between multiple communities. Leverage proved to yield information that was not captured by degree, betweenness, or eigenvector centrality and was more accurate at identifying neighborhood hubs. We propose that this metric may be able to identify critical nodes that are highly influential within the network.

## Introduction

Network theory has recently gained recognition as a useful framework in which to consider the brain in terms of its structure and function. In network analyses of functional magnetic resonance images (fMRI), each voxel can be treated as a node in a network with connections between nodes defined by functional activity [1], [2], [3]. Although two foci in the brain may not have a direct neuronal connection, a functional connection may be inferred based on fMRI time signal correlations [4]. The focus of the outcomes from such an analysis is on the interconnections between areas rather than on the areas themselves. An advantage of using network theory methodologies over traditional fMRI analyses is that the brain is treated as an integrated system rather than a collection of individual components [5]. In addition, network analyses can simultaneously characterize properties of the network as a whole as well as the role each node plays in the network.

Among the many nodes that form a network, some play a crucial role in mediating a vast number of network connections. Such nodes are central in network organization, and are often identified by quantities known as centrality metrics [6], [7], [8], [9], [10], [11], [12], [13], [14]. These centrality metrics identify nodes that are likely to be highly influential over the behavior of the network and are in the mainstream of information flow. One such metric defines central nodes to be those having the highest number of connections, or degree, and is known as degree centrality [7]. This centrality metric assumes that the importance of a node in the network is dictated by the number of other nodes with which it directly interacts. While node degree often proves to identify critical network elements [15], a highly essential node in the brain network may not necessarily have ubiquitous connections to other nodes in the network as assumed by degree centrality.

An increasingly popular centrality metric, eigenvector centrality [12], is unique in that it considers the centrality of immediate neighbors when computing the centrality of a node. Mathematically, eigenvector centrality is a positive multiple of the sum of adjacent centralities [13], and is based on the philosophy that a node is more central if its neighbors are also highly central. However, eigenvector centrality does not account for the disparity in the degree of a node with respect to its neighbors, which has different implications depending on the network's assortativity, or the tendency for nodes to be connected to similar degree nodes. Furthermore it is computationally intensive as compared to other centrality metrics.

Betweenness centrality [11] considers nodes along the shortest geodesic paths to be the most central in the network. In the context of a social network, a person has high betweenness centrality if they are strategically located as middlemen between several pairs of people and, therefore, control the flow and integrity of information between those people. Betweenness centrality assumes that information travels through a network along the shortest path in a serial fashion (see however [16]). Despite the potential utility of this measure of centrality, it is not ideal for a system that processes information via unrestricted walks. For example, in distributed processing systems without a central controller, such as the brain, information typically does not follow shortest paths as they are not predetermined. In the terminology introduced by Stephen Borgatti [17], flow in a network occurs through transference, serial transmission, or parallel duplication. In addition, the flow can utilize a walk, a trail, a path, or a geodesic (shortest path). Parallel duplication following a walk occurs when a single node (such as a neuron or pool of highly correlated neurons) passes information to multiple other nodes simultaneously. Such a system not only utilizes the shortest path but sends information along all possible paths. While not the most efficient method of information transfer, such a process increases the probability that a signal reaches the intended destination. This is particularly true for dynamic systems, like the brain, where existing connections can become impassable or where new connections may become active. Much like the spread of a disease in a social network, we propose that brain networks most likely process information via parallel duplication along unrestricted walks. In other words, information can be passed to multiple neighbors (parallel routes), is not lost by the sender (duplication), and is not restricted along geodesics or paths (unrestricted walks).

Although degree, betweenness, and eigenvector centrality are three of the most widely used measures, there are many others. Closeness centrality [11] is the mean distance between a node and all other nodes in a graph. Subgraph centrality [18] rates the importance of a node based on the number of closed walks beginning and ending at a particular node. These closed walks are weighted based on length, such that the shortest walks contribute the greatest towards the centrality value. The concept of local leaders [19], while not introduced as a centrality metric, captures information similar to centrality. Local leaders are nodes having a degree equal to or greater than all neighbors, and strict local leaders are nodes having a degree strictly greater than all neighbors. Although a myriad of centrality measures exist, the focus of this study has been directed to the analysis of degree, betweenness, and eigenvector centrality as they are the most commonly used centrality metrics in brain networks.

This work proposes a new centrality metric called *leverage centrality* that is designed to identify critical network nodes. Leverage centrality considers the degree of a node relative to its neighbors and operates under the principle that a node in a network is central if its immediate neighbors rely on that node for information. As a social network example, the most popular teenager in a clique can easily shape current fashion trends if her friends do not receive fashion opinions from many other people. Leverage centrality captures nodes in the network which are connected to more nodes than their neighbors and, therefore, control the content and quality of the information received by their neighbors. Leverage is designed to capture the local assortative or disassortative behavior of the network, as node degree is evaluated with respect to degrees of immediate neighbors. It is key to note here that although leverage is derived from degree centrality, there is a distinct difference between the two. A high degree node is not highly central according to leverage if all of its neighbors are also high degree. Furthermore, leverage centrality does not assume information flows along the shortest path or in a serial fashion, but rather focuses on the disparity in node degrees in a small neighborhood to quantify consolidation and dissemination of information locally. Leverage is defined on the interval (−1, 1), making inter- and intra-network comparisons straightforward. Furthermore, calculating leverage centrality is not computationally burdensome, and as such can easily be computed for networks containing on the order of 10^4^ nodes or more.

Nodes identified through leverage centrality are critical for the function of the global network as well as local communities of network nodes known as modules [20], [21], [22], [23]. Many networks, and in particular brain networks, have demonstrated hierarchical structure and may be decomposed into modules or neighborhoods of nodes which perform similar processes [20], [22], [23], [24]. Each module consists of several nodes having a relatively high number of connections within the module compared to the number of connections to nodes in other modules. Leverage centrality may be of particular use in such hierarchical networks as an aid in identifying hubs, nodes that are important to maintaining local topological structure. A hub is the best connected node within the module and, therefore, is likely to have high leverage centrality since its degree is high with respect to other nodes in the neighborhood.

To investigate the utility of leverage centrality in the brain network, we analyzed healthy human brain networks generated from fMRI data using leverage, degree, betweenness, and eigenvector centrality, and we characterized the relationship between these centrality metrics. The spatial distribution of high leverage nodes throughout the brain was examined to gain further insight into the role of high leverage nodes in information distribution. Finally, leverage was evaluated in terms of its ability to detect hubs in the brain network using functional cartography methods [21].

## Methods

### Ethics Statement

This study included 10 volunteers (average age 27.7 years, standard deviation 4.7 years) representing a subset of a previous study [25]. The study protocol, including all analyses performed here, was approved by the Wake Forest University School of Medicine Institutional Review Board. All subjects gave written informed consent in accordance with the Declaration of Helsinki.

### Network Generation

Networks were generated using fMRI time series data from each subject. Gradient echo EPI images (TR/TE = 2500/40 ms) were acquired over a period of 5 minutes at rest (120 images) on a 1.5 T GE twin-speed LX scanner with a birdcage head coil (GE Medical Systems, Milwaukee, WI). Images were corrected for motion, normalized to the MNI (Montreal Neurological Institute) space, and re-sliced to 4×4×5 mm voxel size using SPM99 (Wellcome Trust Centre for Neuroimaging, London, UK).

**Figure 1:**
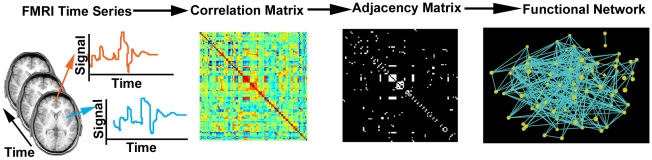
The process of generating functional networks. Resting state fMRI data are collected from a subject. Voxel time series are extracted from the set of images, and a Pearson correlation analysis is performed between all possible pairs of voxels. The correlations are represented in the form of a correlation matrix, which is binarized at a given threshold to yield an adjacency matrix. The functional network is thereby defined, where each voxel is represented by a node and connections are determined by the adjacency matrix.

Network generation is depicted in Figure 1. Time courses were extracted from each of approximately 16,000 voxels corresponding to gray matter areas in normalized brain space and corrected for physiological noise by band-pass filtering to eliminate signal outside of the range of 0.009–0.08 Hz [2], [4]. Mean time courses from the entire brain (the average of voxel values within the brain parenchyma mask including gray and white matter), the deep white matter (average time course in an 8 mm radius sphere within the anterior portion of the right centrum semiovale composed entirely of white matter), and the ventricles (average of time courses within the ventricle mask created by the WFU PickAtlas Tool [26]) were regressed from the filtered time series. The six rigid-body motion parameters from the motion correction process were also regressed out from the time series.

A correlation matrix was populated by computing the Pearson correlation between all possible pairs of the 16,000 voxels. A threshold was applied to the correlation matrix, above which individual voxels were said to be connected, thereby discretizing the correlation matrix into a binary adjacency matrix with values of 1 indicating the presence and values of 0 indicating the absence of a connection between two voxels. The threshold was defined such that the relationship between the number of nodes and average number of connections between nodes was consistent across subjects. Specifically, the relationship 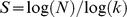 was the same across subjects, where *N* was the number of nodes in the entire network, *k* was the average degree of the network, and *S* represented the average path length of an Erdõs-Rényi network [27]. In this work, we chose S = 3.0 as the threshold to define networks, but network properties have been demonstrated to be robust for different S values [3].

Equivalent synthetic random networks were generated by randomly rewiring networks in a fashion similar to that described in [28]. Specifically, nodes were rewired so that the degree distribution remained unchanged but network connectivity became randomized. The three centrality metrics were compared between the original networks and their equivalent synthetic networks. This allowed for the comparison of each brain network to a null condition, where the degree distribution of the network was held constant but any assortative behavior or other topological properties particular to the organization of the brain network were removed.

### Centrality Computations

Leverage (l*_i_*), degree *(k_i_)*, betweenness *(b_i_)*, and eigenvector centrality *(e_i_)* were calculated for each node of the 10 brain networks and their equivalent synthetic networks. Degree was determined by the number of neighbors connected to node *i*. Betweenness was defined by the equation below, where *g_xy_* is the number of shortest geodesic paths between any two nodes *x* and *y*, and *g_xiy_* is the number of those geodesics passing through node *i*.
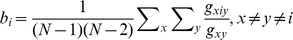
Eigenvector centrality was calculated according to the equation below, where λ denotes the largest eigenvalue and *e* denotes the corresponding principal eigenvector.
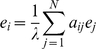
From the above equation, the eigenvector centrality *e_i_* of a node *i* is given by the sum of the values within the principal eigenvector *e* corresponding to direct neighbors, as defined by the adjacency matrix (i.e. where *a_ij_* ≠ 0). Eigenvector centrality is then scaled by the proportionality factor 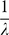. In a discussion on normalization of eigenvector centrality, Ruhnau [13] has shown that Euclidean normalization produces an eigenvector centrality that can attain a maximal value of 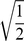 regardless of network size. By multiplying the resulting eigenvector centrality values by 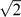, the maximum achievable value becomes 1, and can be attained only by a node at the center of a star. Additionally, since only the largest eigenvalue and corresponding eigenvector must be obtained, a power iteration algorithm was implemented to increase computational efficiency as recommended by Lohmann et al. [29].

Leverage centrality is a measure of the relationship between the degree of a given node (*k_i_*) and the degree of each of its neighbors (*k_j_*), averaged over all neighbors (*N_i_*), and is defined as shown below.
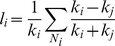



A critical aspect of this computation is that the degree of node *i* is not simply compared to the average degree of its neighbors. Because the degree distributions of brain networks have been shown to be either exponentially truncated power laws [3], [5], [30], [31], [32] or scale-free distributions [1], [2], highly connected nodes can significantly skew the average. A node with negative leverage centrality is influenced by its neighbors, as the neighbors connect and interact with far more nodes. A node with positive leverage centrality, on the other hand, influences its neighbors since the neighbors tend to have far fewer connections.

### Correlation Analyses

Correlation analyses were used to explore the relationships between the four centrality metrics. The examination of different centrality metrics for each node in the brain network allowed for a comparison of the similarity or dissimilarity of each method. Scatter plots of the degree, leverage, betweenness, and eigenvector centrality were created for the brain networks of all subjects. The correlations among the node-wise centrality metrics were calculated for each subject. Resulting brain overlap images were visualized using MRIcro (http://cnl.web.arizona.edu/mricro.htm).

### Modularity Analyses

Modularity analyses were run on each subject, utilizing the QCUT algorithm developed by Ruan and Zhang [33]. This modularity algorithm parcellates each functional network into modules or communities of nodes that are more interconnected among themselves than they are connected to the rest of the network. The presence of these highly interconnected communities has been termed “community structure” [14], [24], [33], [34], [35]. Modularity is an NP hard computational problem [34] and thus requires algorithms that approximate the solution. Various methods for identifying network substructure have previously been reviewed [36]. Most methods use Modularity (quantified by the parameter Q) [35] to identify the optimal network subdivision. This variable compares the number of intermodular edges in the divided network to the number of intermodular edges in a random network with the same subdivisions. Q ranges from 0 to 1 with higher values indicating greater modular organization. In real networks, values of Q typically do not greatly exceed 0.7.

Since all functional networks are unique, the network parcellation for each subject is unique. The Jaccard index was used as a measure of similarity between subjects to identify the most representative subject in the study [33]. For two subjects *x* and *y* with modular divisions *M_x_* and *M_y_*, the comparison between modularity results were computed as
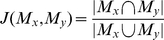



The Jaccard index is the ratio of the intersection of the classification of the two modular structures divided by the union [37]. The Jaccard index between two subjects is high if the community structures are very similar. A similarity matrix was generated to compare all subjects, and a total similarity index was generated for each subject by summing all Jaccard indices computed for a given subject. The most representative subject was that with the highest total similarity index [24].

The QCUT algorithm was chosen to identify network modularity as we have found QCUT to be very robust and highly reproducible for identifying an optimal network division based on Q. In an analysis of this algorithm (see Text S1, Figure S1, and Table S1) a particular brain network was divided into the modular organization in 15 independent runs. The resulting parcellations were highly reproducible with a mean Jaccard index of 0.93 (SD 0.018). This indicates highly reproducible subdivisions that exhibited trivial differences. In particular, the 9 runs that generated the highest Q value (0.673) all had Jaccard indices of 0.945.

### Network Hubs

A method of identifying and classifying hubs in networks that considers neighborhood structure, introduced as functional cartography, was established by Guimera and Amaral [21] and adopted by others [24], [38]. This method compares the participation coefficient *pc_i_* to the normalized within-module degree *z_i_*. The participation coefficient captures the distribution of the links of a node. If a node has equal links to all of the modules of a network, its participation coefficient approaches 1. However, if all links belonging to the node lie within its own module its participation coefficient is 0. The participation coefficient for a node *i* belonging to a module *m* in a network with 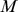 total modules is computed as
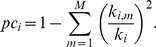



**Figure 2:**
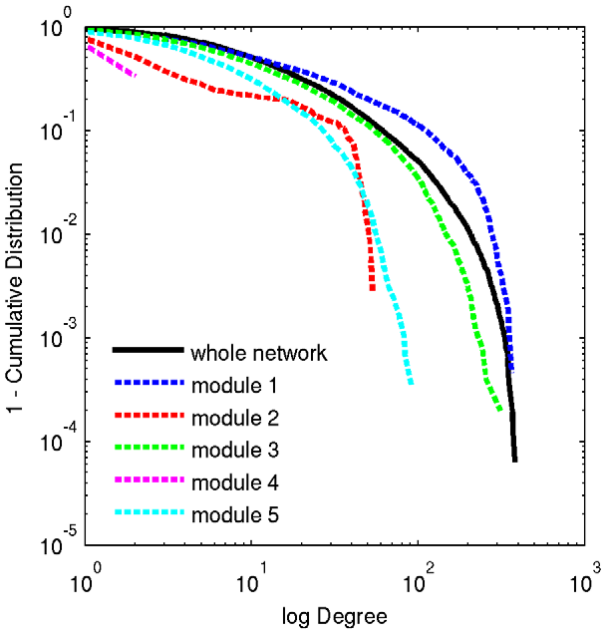
Functional brain networks follow an exponentially truncated power law degree distribution. Degree distributions of the whole network (solid line) and individual modules for a representative subject (subject 5). All modules as well as the whole network follow an exponentially truncated power law distribution.

The term *k_i,m_* denotes the within module degree, or the number of connections between node *i* and other nodes within module *m*, and *k_i,m_/k_i_* indicates the ratio of connections a node has within its own module. Often a normalized z-score with the mean and standard deviation of the within module degrees is used to describe within module connectivity, assuming the node degrees have a normal distribution [21]. However, the degree distribution in brain networks is more closely approximated by an exponentially truncated power law distribution [3], [5], [30], [31], [32]. (It is noteworthy that others have shown that brain networks may approximate a scale-free distribution [1], [2], but networks analyzed in our laboratory have been in support of the exponentially truncated power law [3].) This is true even for within module degree distributions as demonstrated in Figure 2 for one representative subject from the study (subject 5). Therefore, we chose to represent the within module degree by the degree p-value *pk_i_* determined by 1 minus the cumulative distribution function (CDF) of within module degrees. The within module degree p-value is given by dividing the number of nodes in a given module with a degree greater than or equal to *k_i_* by the total number of nodes in the module.

**Figure 3:**
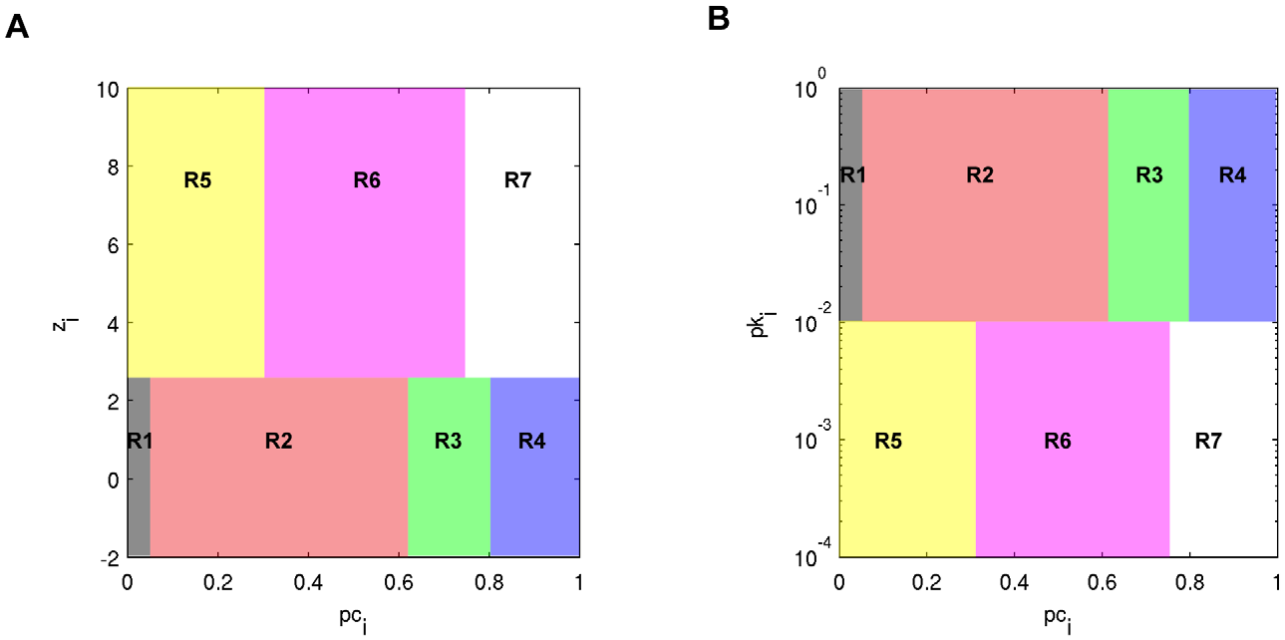
Comparison of *pc-z* space versus *pc-pk* space. (A) Within-module degree z-score *z_i_* and participation coefficient *pc_i_* are used to designate nodes into seven regions as described in [21], [24], [38]. Nodes are designated as hubs if *z_i_*≥2.5 and non-hubs otherwise. Regions are defined as: R1 – ultra-peripheral nodes; R2 – peripheral nodes; R3 – non-hub connector nodes; R4 – non-hub kinless nodes; R5 – provincial hubs; R6 – connector hubs; R7 – kinless hubs. (B) Within-module degree probability *pk_i_* and participation coefficient *pc_i_* are used to designate nodes into the seven regions defined above. Participation coefficient classifications are identical to (A), but the cutoff *pk_i_*≤0.01 is used to define hubs versus non-hubs, corresponding to *z_i_*≥2.5 when approximating with a normal distribution.

Nodes with high degree *k_i_* have a low p-value *pk_i_* since there are relatively few hubs in networks with an exponentially truncated power law distribution, where the highest degree nodes, or hubs, are far less likely to occur than non-hubs. For this reason, hubs were classified as being those nodes having a within module degree probability less than 0.01. This criterion is analogous to having a z-score above 2.5 as suggested by Guimera and Amaral [21], corresponding to p<0.01 in a Gaussian distribution. Hub nodes (*pk_i_*≤0.01) were further delineated into provincial, connector, or kinless hubs by the participation coefficient in accordance with [21]. Hubs having *pc_i_*≤0.3 were said to be provincial hubs, as their low participation coefficient reveals that they are extremely well connected within their own module. Connecter hubs were those hubs having 0.3<*pc_i_*≤0.75, indicating that they served to connect nodes in other modules to their own module. Kinless hubs had participation coefficient values *pc_i_*>0.75, indicating that almost all of their neighbors are distributed in other modules. No kinless hubs were found in any of the 10 networks analyzed and are not discussed further. Figure 3 illustrates the similarity between *pc-z* space versus *pc-pk* space. The advantage of using the *pk* form of within module degree is that a degree distribution is not assumed. However, for small networks it may be difficult to find a node with sufficiently small pk. In such cases *pc-z* space is the more appropriate method.

Degree, leverage, betweenness, and eigenvector centrality were examined as an additional axis to a functional cartography plot to compare the metrics' abilities to identify hubs in brain networks. Nodes were classified as hubs based on varying a cut-off criterion for each centrality metric. If the node centrality was greater than a given criterion, the node was classified as a hub. By changing the criteria for degree, betweenness, eigenvector, or leverage centrality over a range of thresholds, the metrics were compared in terms of accuracy in identifying network hubs with results determined by functional cartography described above. Centrality criteria were equally spaced in 10000 increments along the range of the respective metric. As an example, Table 1 provides threshold criteria used for each method for subject 5.

**Table 1:** Example of threshold values used in generation of ROC curves.

	Minimum	Interval	Maximum
Leverage	−0.9873	1.5509 e-04	0.5634
Degree	1	0.0381	382
Betweenness	0	4.6419 e-07	0.0046
Eigenvector	0	1.0306 e-05	0.1030

Since the true hub classification is not known for brain networks, functional cartography was utilized as an alternate to centrality measures. While functional cartography does not provide a definitive or “gold standard” hub classification, it is a well-studied method [21], [24], [38] that does not rely solely on the number of connections (degree) to identify hubs. This method also allows for the identification of hub structure within and between network neighborhoods. Although the cartography method is dependent on the modularity analysis used, the QCUT algorithm used to define the modular structure is highly reproducible, as discussed previously. However, to be thorough, we acknowledge that the high precision of the QCUT algorithm does not ensure the accuracy of the functional cartography results. A full evaluation of the accuracy of neighborhood hub detection using cartography and leverage centrality based on known networks is beyond the scope of this paper.

The true positive and false positive rates were calculated to yield receiver operating characteristic (ROC) curves for each subject. True positives were classified as nodes that were defined as hubs using functional cartography (*pk_i_*≤0.01), which were also classified as hubs based on the centrality criterion. False positives were those nodes which were not defined as hubs using functional cartography (*pk_i_*>0.01) but classified as hubs based on the centrality criterion. The area under the curve (AUC) for each ROC was computed for each centrality metric and compared in all subjects using multiple pairwise t-tests with Bonferoni correction to test for differences between the centrality metrics.

## Results

### Correlation Analyses

**Figure 4:**
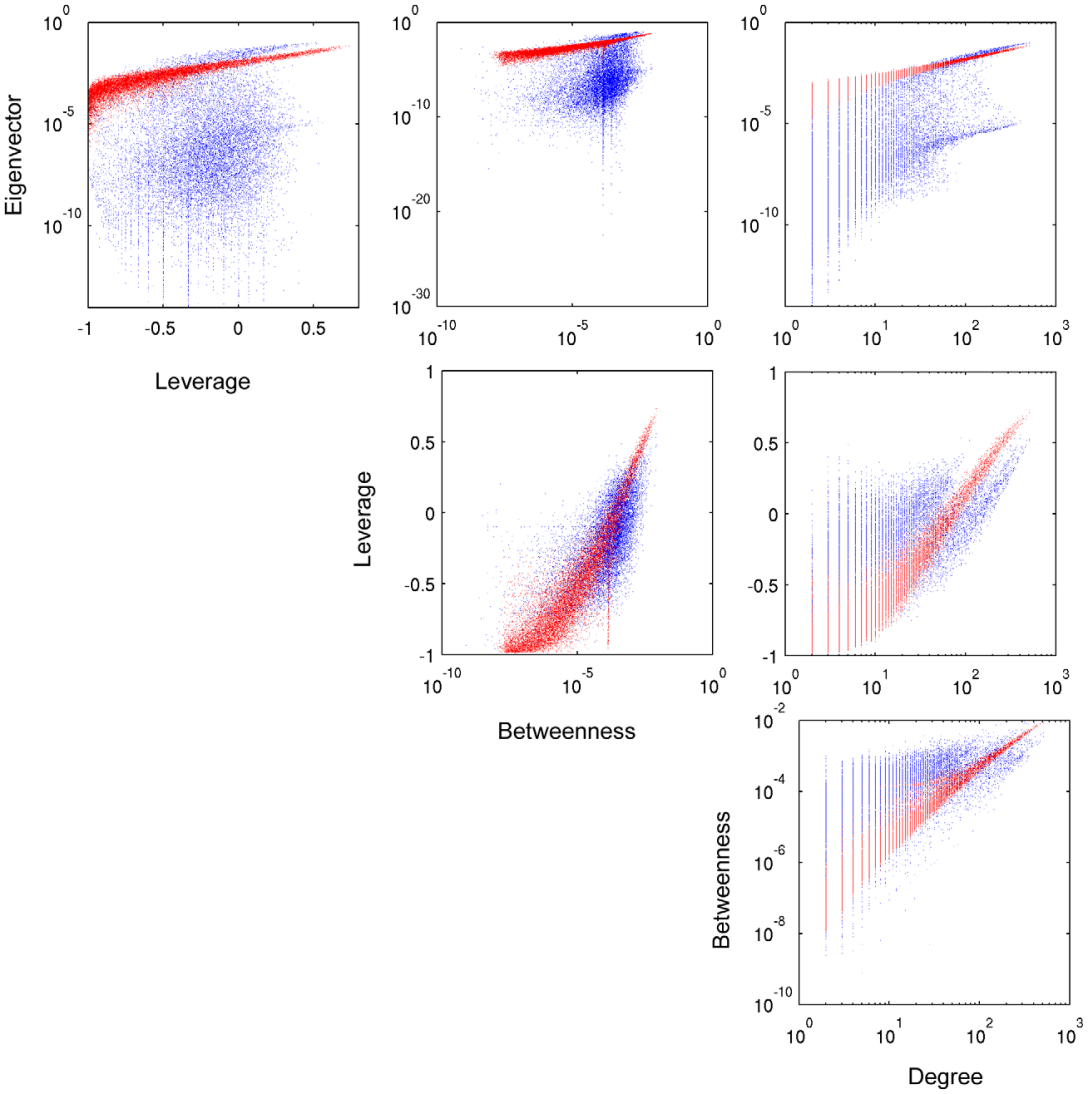
Scatterplot matrix of leverage, degree, betweenness and eigenvector centrality for the brain network of a representative subject. Labels to the left of plots indicate the ordinate centrality, where labels beneath plots indicate the abscissa centrality. Synthetic network nodes (red) overlaid over the original network (blue) separate nodes from the original network into distinct groups, most notably in plots involving leverage or eigenvector centrality.

Correlation plots of the centrality metrics for a representative subject are shown in the scatter plot matrix in Figure 4. Within the scatter plot matrix each centrality metric is indicated along the diagonal such that, for any given plot, the abscissa is specified by the label in the lowest row, and the ordinate is specified by the label in the left-most column. These plots reveal the relationships between leverage, degree, betweenness, and eigenvector centrality. However, while all centrality metrics are positively correlated, there is not a strict linear relationship in any of the cases. Correlations between eigenvector centrality and either leverage or betweenness centrality are noticeably lower than the correlation between eigenvector centrality and degree centrality. Although leverage and eigenvector centrality are both derivatives of degree centrality, clearly these metrics do not convey the same information.

**Figure 5:**
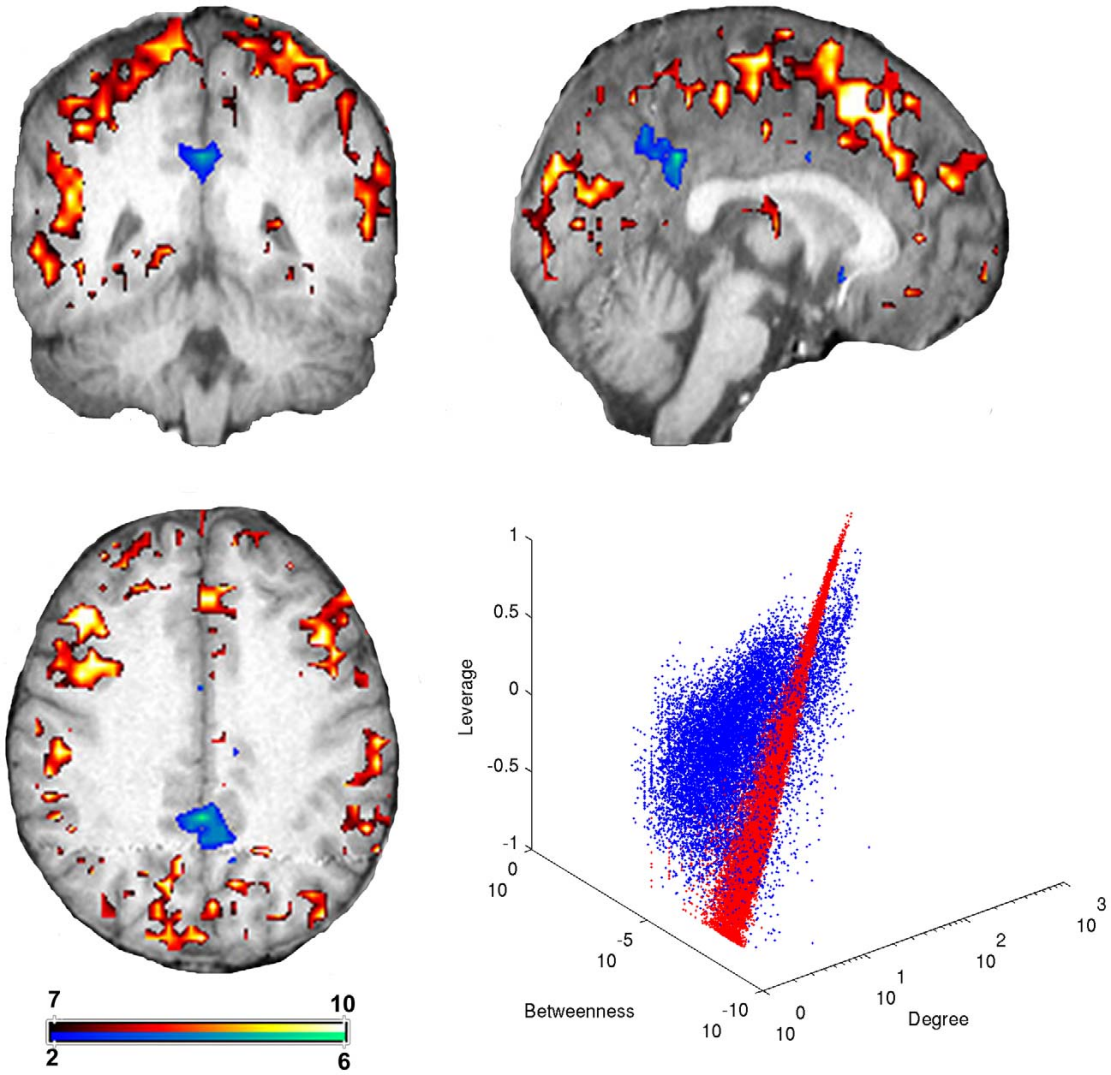
Overlap image compiled from all subjects. Intensity values correspond to the number of subjects having a particular network node, i.e. image voxel, above (warm colors) or below (cool colors) the synthetic network degree-leverage centrality scatter plot. Nodes below the synthetic distribution, primarily concentrated in the areas of the precuneus and posterior cingulate, are highly interconnected high degree nodes with many redundant connections. Nodes above the synthetic distribution have higher leverage than synthetic network nodes with the same degree and can be found scattered throughout the gray matter. Color bar represents the number of subjects that exhibited a node in any particular location.

One of the more intriguing qualities of the centrality metrics is the apparent grouping of nodes in the scatter plots, particularly in those including leverage and eigenvector centralities. Two clearly distinct groups of data are most evident in the 3-dimensional plot of leverage, degree, and betweenness, separated by points from the synthetic network (Figure 5). The same occurrence is seen in all subjects. Interestingly, the synthetic network data overlaid over the original network data have much stronger linear relationships. Correlation values between centrality metrics in the original and synthetic networks are displayed in Table 2.

**Table 2:** Correlation between centrality metrics averaged across 10 subjects, +/− standard deviation.

	Original Network	Synthetic Network
Leverage vs. Degree	0.518+/−0.072	0.842+/−0.020
Leverage vs. Betweenness	0.590+/−0.066	0.646+/−0.035
Leverage vs. Eigenvector	0.170+/−0.032	0.720+/−0.089
Betweenness vs. Degree	0.621+/−0.119	0.931+/−0.008
Betweenness vs. Eigenvector	0.204+/−0.075	0.917+/−0.006
Degree vs. Eigenvector	0.643+/−0.101	0.994+/−0.001

To further investigate this phenomenon, nodes above and below the synthetic network distribution were identified in brain space in each subject. A single overlap image was created (Figure 5) indicating consistent spatial patterns of nodes above or below the synthetic network distribution for all subjects. This image was created by summing the number of subjects that had a particular voxel above (warm colors) or below (cool colors) the synthetic data.

The network nodes with the highest degree centrality typically fell below the synthetic network nodes in Figure 5. The loss of such individual nodes from this group would not greatly impact the topology of the network since they are highly interconnected with redundant connections. These nodes were largely centered in the posterior cingulate and precuneus, regions of the brain previously shown to be the core of the anatomical brain network [39] and known to be highly interconnected [3]. This high level of local interconnectedness caused this region to be classified as low leverage with respect to the synthetic network. Therefore, nodes in this population are less influential than would be predicted for nodes of similar degree centrality in a randomly connected network.

Nodes above the synthetic network nodes in Figure 5 are connected to lower degree nodes and have higher leverage than synthetic network nodes with the same degree. Interestingly, these nodes appeared to be dispersed throughout the cortex. Such a dispersion of high leverage nodes may contribute to efficient information diffusion throughout the brain network.

**Figure 6:**
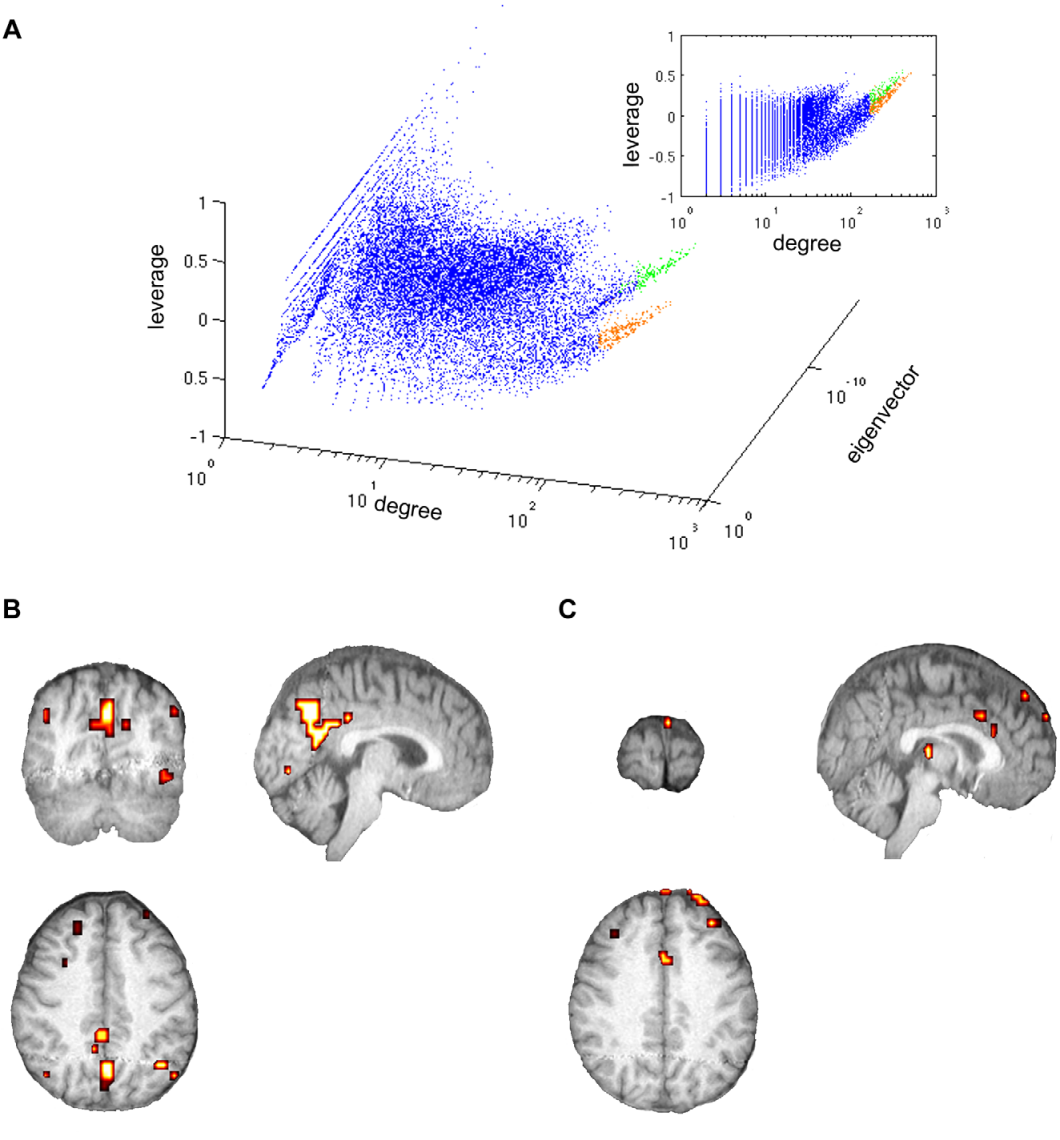
Eigenvector centrality reveals additional network subgroups. (A) Scatter plot of leverage, degree, and eigenvector centrality, where the lower group of nodes observed previously is shown to consist of two subgroups with different eigenvector centralities. Inset shows that the subgroup with higher eigenvector centrality (orange) has slightly lower leverage centrality than the subgroup with lower eigenvector centrality (green). (B) Spatial distribution of subgroup with higher eigenvector centrality but slightly lower leverage centrality (orange subgroup). (C) Spatial distribution of subgroup with lower eigenvector centrality but slightly higher leverage centrality (green subgroup).

A 3-dimensional plot of eigenvector, leverage, and degree centrality revealed that the group of nodes below the synthetic network data in Figure 5 in fact consisted of two subgroups (Figure 6A). In this figure, the synthetic network data have been omitted for simplicity, but the divisions originally noted in Figure 5 are still clearly distinguishable. Two subgroups of the nodes below the synthetic network were separated by high or low values of eigenvector centrality. The first subgroup (highlighted in orange) was concentrated at low values of eigenvector centrality, while the second subgroup (highlighted in green) was concentrated at higher values of eigenvector centrality. Interestingly, the inset of leverage vs. degree shows that the subgroup with lower eigenvector centrality (green) in fact had slightly higher leverage centrality. Nodes belonging to the green subgroup are connected to lower degree nodes than themselves, and therefore eigenvector centrality is slightly reduced while leverage is slightly elevated. Conversely, the subgroup with higher eigenvector centrality (orange) consists of nodes where the disparity between the degree of a given node and of the neighbors is less pronounced than in the green subgroup. Therefore, in this subgroup the leverage centrality is slightly reduced, while the eigenvector centrality is slightly elevated.

The spatial distributions of subgroups are shown in Figure 6B (orange subgroup) and Figure 6C (green subgroup). Nodes from the orange subgroup, having slightly elevated eigenvector centrality and slightly reduced leverage centrality were found largely in the region of the precuneus and posterior cingulate. Nodes from the green subgroup, with slightly elevated leverage but slightly reduced eigenvector centrality, were distributed in the prefrontal cortex, anterior cingulate, and thalamus. Although results are presented for a single subject, these patterns are representative of results from all subjects as demonstrated in the supplemental materials (see Text S1 and Figure S2).

### Leverage as a Detector of Module Hubs

**Figure 7:**
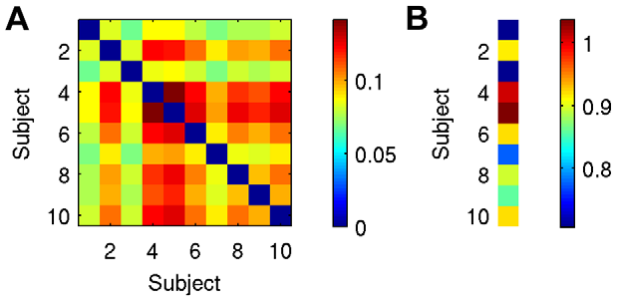
Results of similarity analysis. (A) Jaccard indices between all possible subject pairs, where the diagonal has been constrained to zero. (B) Sum of Jaccard indices for each subject, revealing subject 5 to have the highest similarity across subjects.

**Figure 8:**
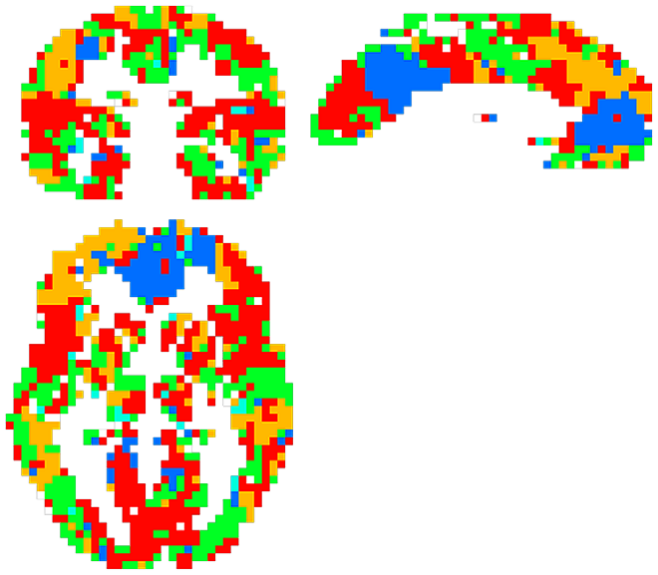
Modules of the brain of a representative subject. Each color corresponds to a particular functional module, with 7 total modules present, in a representative subject (subject 5).

Module parcellation was performed using the QCUT algorithm and resulted in a unique definition of community structure for each subject. Similarity between subjects, measured via the Jaccard index, revealed subject 5 to be the most representative (Figure 7). Modularity results for the most representative subject are shown in brain space, where each module is represented by a different color (Figure 8). As can be seen from Figure 8, although modules were not necessarily spatially contiguous, they tend to spatially cluster in different regions of the brain.

**Figure 9:**
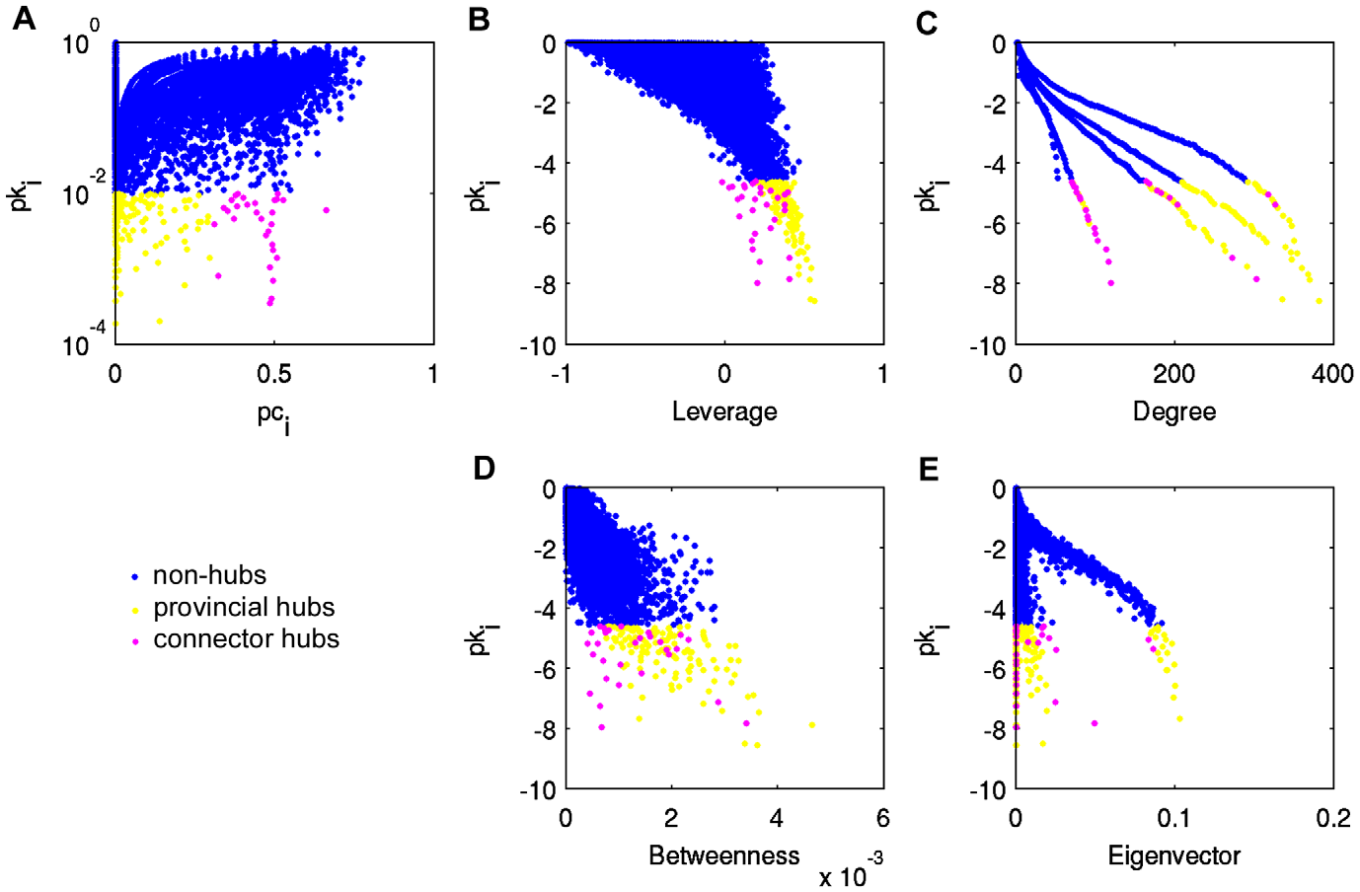
Extension of functional cartography. (A) Functional cartography plot of brain network from subject 5. Within module degree probability *pk_i_* is shown versus participation coefficient *pc_i_*. Hubs are delineated as provincial (yellow) or connector (pink) based on thresholds defined in the text. The functional cartography plot has been extended to include leverage (B), degree (C), betweenness (D), and eigenvector centrality (E) of the same network.

Functional cartography plots were generated to identify provincial and connector hubs (Figure 9A). Nodes that had within-module degree probabilities (*pk_i_*) less than 0.01 were delineated as hubs. All hubs were then defined to be either *provincial hubs* that are key to the structure within their native module, or *connector hubs* that serve to link multiple modules. The assignment to either provincial or connector hubs was based on the participation coefficient (*pc_i_*) thresholds defined in the methods. Each plot was extended to include leverage centrality (Figure 9B), degree (Figure 9C), betweenness (Figure 9D), or eigenvector (Figure 9E) centrality on a 3^rd^ axis. Interestingly, connector and provincial hubs were distributed throughout the ranges of degree, betweenness, and eigenvector centrality, but were concentrated at higher values of leverage. In other words, leverage centrality was capable of providing a reasonable cutoff, above which nodes may be classified as either hubs or non-hubs. In contrast, since the hubs spanned the entire range of the other three centrality metrics, there was no clear threshold in either case above which solely hubs existed.

**Figure 10:**
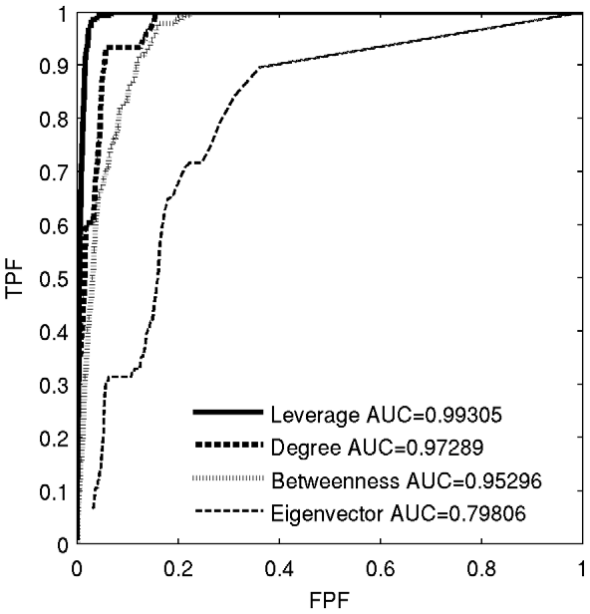
Receiver Operating Characteristic curves for a representative subject. ROC curves reflect the higher accuracy of hub detection using leverage, degree, betweenness, or eigenvector centrality. In this case the representative subject (subject 10) had AUCs closest to the mean. Results are typical of all but one subject, where degree was found to be the most accurate method.

ROC analyses (Figure 10) are presented to better show the greater potential for classification of hubs using leverage over the other metrics. As discussed in greater detail in the Methods section, ROC analyses were used to evaluate the accuracy of each centrality metric in identifying and classifying hubs as compared to the results of the functional cartography analyses. These ROC analyses revealed leverage to be the most accurate hub detector in all but one subject. On average, leverage ROC curves had the highest average AUC (0.99+/−0.01) as compared to degree (0.97+/−0.02), betweenness (0.96+/−0.02), or eigenvector centrality (0.75+/−0.08). In a subject that did not fit this pattern, degree had the greatest AUC (0.97346) over leverage (0.97026), betweenness (0.91549), or eigenvector centrality (0.69819).

**Figure 11:**
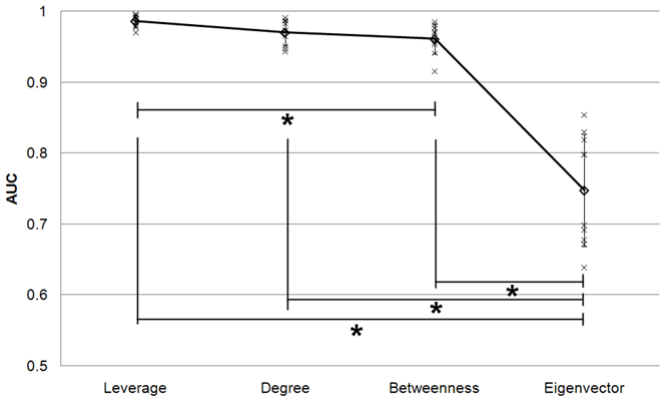
AUCs for ROC curves for identifying hubs in all subjects. AUC values demonstrate the accuracy of detecting hubs using leverage, degree, betweenness, or eigenvector centrality. Trend (average - diamonds) shows that the highest average AUC is for leverage centrality ROC curves. Asterisks indicate statistical significance (p<0.05).

Multiple pairwise t-tests with Bonferoni correction revealed significant differences in AUCs between leverage and betweenness (p = 0.005), between leverage and eigenvector (p<0.001), between degree and eigenvector (p<0.001), and between betweenness and eigenvector (p<0.001), marginal significance between leverage and degree (p = 0.06), but no significant differences between degree and betweenness (p>0.999). Leverage, having significantly higher mean AUC than the other metrics, was shown to be the most effective at identifying hubs, i.e. nodes which play a critical role in community structure (Figure 11).

**Figure 12:**
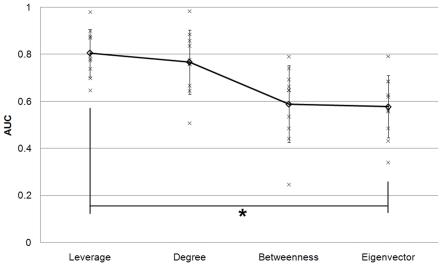
AUCs for ROC curves for classifying hubs in all subjects. AUC values compare the accuracy of distinguishing between provincial and connector hubs using leverage, degree, betweenness, or eigenvector centrality. Trend (average - diamonds) shows highest AUC is for leverage centrality ROC curves. Asterisk indicates statistical significance (p<0.05).

In addition to the concentration of hubs at higher values of leverage centrality seen on the functional cartography plots (Figure 9B), a distinct clustering of connector hubs versus provincial hubs can be observed. On cartography plots with leverage plotted on the 3^rd^ axis, connector hubs were found at less extreme values of leverage (*l_connector_* = 0.2318+/−0.1228) than provincial hubs (*l_provincial_* = 0.3479+/−0.0688) for all subjects. In the cases of degree, betweenness, and eigenvector centrality, both hub types appeared to span the range of the respective centrality metric. The ability of the metrics to distinguish between provincial and connector nodes was evaluated using ROC curve analysis considering only the provincial and connector hubs. The same criteria for degree, betweenness, and leverage were employed as in the previous analysis. The AUC of ROC curves corresponding to leverage had the highest mean (0.81+/−0.10) as compared to degree (0.77+/−0.14), betweenness (0.59+/−0.16), and eigenvector centrality (0.58+/−0.13) (Figure 12). Similar to the previous analysis, multiple pairwise t-tests with Bonferoni correction were performed to test for differences in the ability of leverage, degree, betweenness, and eigenvector centrality to distinguish between connector and provincial hubs. The analysis revealed a significant difference between leverage and eigenvector centrality (p = 0.021), while the difference between degree and eigenvector centrality was only marginally significant (p = 0.06). The differences in AUC were not significant between leverage and degree (p>0.999), leverage and betweenness (p = 0.10), degree and betweenness (p = 0.30), or betweenness and eigenvector centrality (p>0.999).

## Discussion

In this work leverage has been introduced as a new metric of network centrality to evaluate the role of individual nodes in brain networks. Leverage is unique from existing centrality methods in that it does not assume serial transportation of information, but rather allows for parallel processing such as that occurring in the brain. Leverage accounts not only for the degree of a given node, but also for the degree of its neighbors, thereby capturing local assortative or disassortative behavior. This has important implications for information transfer and the influence one brain region may have over another. Individual brain cells accumulate information from all active synapses and integrate this information over space and time, and if the total signal surpasses a set threshold the neuron will fire. Since any given input to a neuron is combined with all other active inputs, its influence is dependent on the number of other active connections. A neuron (X) that synapses with many other neurons that each only has a few inputs will be highly influential over that population of cells. In other words neuron X will have high leverage. On the other hand, if a different neuron (Y) synapses with many neurons that all have many inputs, neuron Y will not be very influential; neuron Y is a low leverage neuron. Of course it is important to note that this discussion is on the level of the neuron but the data presented here was from networks generated at the level of the voxel – many orders of magnitude larger than the neuron. While it can be argued that behavior at the neuronal level may propagate to the voxel level, the most appropriate scale has been difficult to ascertain [40], [41].

Development of new measures of centrality must consider the computational burden of the metric. Some measures can have computational costs that are too high to be useful for large networks. Importantly, the computation for leverage is inexpensive in terms of CPU load. As an example, it took 9.6 seconds to compute the leverage centrality for a network with *N* = 14,323 nodes and average degree *k* = 27 on a RedHat Linux workstation with a 3.0 GHz Intel Core2 Quad processor with 8.0 GB RAM using Matlab R2008b (MathWorks, Natick, MA). This is compared to the 227.7 seconds required to perform the betweenness calculation and 11.8 *minutes* to perform the eigenvector centrality calculation on the same network. Since leverage is of O(N) (i.e. scales linearly with network size), increasing the network size has little effect on the computational load. Betweenness and eigenvector centrality, on the other hand, are both far more computationally expensive, resulting in sizeable increases in computation time as the network size increases.

We have examined the relationships between leverage centrality and three other well-characterized centrality metrics. Although all centrality metrics were positively correlated, leverage and eigenvector both provided additional information not evident from degree or betweenness alone. This was particularly true when examining networks against synthetic random networks with identical degree distributions. 3-dimensional plots of leverage, degree, and betweenness have revealed the separation of network nodes into two easily recognizable groups divided by synthetic network data. This separation arises as a result of the assortative nature of brain networks. In assortative networks, high degree nodes preferentially connect to other high degree nodes, and likewise low degree nodes tend to connect to other low degree nodes. Since leverage is designed to capture the similarity or dissimilarity in degree between a node and its neighbors, examining leverage made this assortative behavior apparent.

Upon examining the spatial distribution throughout the brain of high and low leverage nodes relative to the random networks, it was shown that nodes falling above the synthetic network (having higher leverage than expected for a node with comparable degree in a random network) were interspersed throughout the brain. However, those nodes below the synthetic network were concentrated in the region of the posterior cingulate and precuneus, a location known to be a core of the brain network [39]. It is interesting to note here that a region considered to be a hub of the brain network in terms of anatomical structure, and one which is a hub in terms of its degree, is not necessarily a hub when considering leverage centrality. Leverage centrality identifies those regions that are not necessarily the most connected ones, but the must influential over immediate neighbors. The posterior cingulate and precuneus regions do not have leverage over the other high degree regions to which they are connected.

A deeper examination of the relationship between leverage and eigenvector centrality allowed for the distinction of two subgroups of data comprising the group of nodes having lower leverage centrality. One subgroup, concentrated at higher values of eigenvector centrality, had slightly lower leverage centrality. On the other hand, the other subgroup, concentrated at lower values of eigenvector centrality, had slightly higher leverage.

Functional cartography was extended to include leverage, degree, betweenness, or eigenvector centrality information as the 3^rd^ axis. The roles of hubs were identified as provincial or connector based on participation coefficients and within module degree probabilities. Leverage was tested for its ability to both classify nodes as hubs or non-hubs and distinguish between connector and provincial hubs using ROC analyses. Leverage proved to be statistically significantly more accurate at detecting hubs versus non-hubs than betweenness or eigenvector centrality, and performed as well as degree in the same task. Leverage also showed promising results in distinguishing between connector and provincial hubs particularly compared to eigenvector centrality. However, the sample size N = 10 subjects used in this study did not provide sufficient evidence to achieve statistical significance in other comparisons. These functional cartography results have shown that high leverage nodes tend to be hubs, and furthermore, the highest leverage nodes tend to be provincial hubs, possibly holding together the modular structure of brain networks. Leverage has therefore been demonstrated to be a viable tool for the identification of hubs in brain networks.

A limitation intrinsic to the study of brain networks is the uniqueness of each subject's functional network. Although preprocessing of the original fMRI time series data attempts to transform the imaging data into a common space across subjects, formation of the brain connectivity network is likely influenced by subject variability. For example, node locations may not perfectly overlap across subjects, and connections defined by correlation coefficients may vary across subjects. Such subtle discrepancies likely result in a network structure that is similar in overall structure [2], [3], [39] but with large local inter-subject variability. Thus, appropriately characterizing properties of centrality metrics in a group can be challenging. Although averaging of the correlation matrices [30] or of centrality metrics across subjects [2], [30] has been considered previously, in our laboratory we have observed a smoothing effect that results in drastic changes in the degree distribution and in modularity (results to be reported elsewhere). As an alternative to averaging, the Jaccard index was used to determine the most representative subject, as the Jaccard index is capable of handling networks of varying sizes. Analyses performed on all other subjects as well as group analyses support the findings in that subject. Processing code for the Jaccard index has been made readily available [42] and is explained in detail in supplementary material [43] from a previous article [33]. However, an analysis method that can capture the overall characteristics of the brain network from a group of subjects is desired in the future.

An additional limitation arises from the alternate hub classification scheme using *pc-pk* space, which does not assume a normal distribution as in the *p-z* space classification method. The disadvantage of pc-pk space is that there is a bias towards detecting hubs even where none exist. Take as an example a module consisting of just 10 nodes, where the average degree is 4. If there is only a single node with a degree of 5 it will have a low p-value (p = 0.01), and pass the criteria for classification as a hub, even though its degree is not such that we would *qualitatively* classify it as a hub. For large enough modules, given a within-module degree distribution following an exponentially truncated or scale-free power law, such a situation is unlikely to occur. However, in smaller modules this is certainly plausible. In such cases, the advantage of the *p-z* space classification scheme is that it takes into account whether a node is a sufficient number of standard deviations from the mean.

Leverage was investigated here in the undirected graphs produced by fMRI data. However, an extension of leverage could easily be applied to directed graphs by computing in-leverage and out-leverage using in-degree and out-degree. Leverage centrality may then be applied to such directed networks as the C. elegans neural network [27], [44], [45], [46], marine [45], [46], [47] or freshwater food webs [45], [46], [48], the world wide web [45], [46], [49], and a multitude of other networks across various disciplines. In such networks, high out-degree nodes may actually have very low out-leverage and, therefore, may not be highly influential over the local behavior of the network. Alternatively, low out-degree nodes may have high-out leverage and be very influential over local network behavior. Detection of high in-leverage and out-leverage nodes may allow identification of components of these networks which are highly important to network structure and stability. For example, high in-leverage nodes in the World Wide Web may be information hubs, sources of information utilized by many locations throughout the network. High out-leverage species in food web networks likely provide nutrition for a large component of the food web, and extinction of these species would significantly undermine the stability of the ecosystem. In such networks as these, leverage could potentially give insight into appropriate preventative measures to protect against network collapse.

In addition to directed networks, leverage may be applied to weighted graphs. In this work, unweighted networks were produced by thresholding the correlation matrix such that the relationship between the number of nodes and number of edges in each network was preserved across subjects. Alternatively, a threshold may be applied across the correlation matrix in order to eliminate spurius connections suggested by low correlations resulting in links that are unlikely to occur in the true network. These low correlations would be replaced by zeros in the adjacency matrix, but sufficiently high correlations would be preserved in order to create the weighted network. Using the weighted equivalent of degree, a weighted counterpart to leverage centrality can then be calculated at each node.

A further possible extension of leverage is to consider the influence of indirect neighbors. In this study, the degrees of 1-hop neighbors were considered in the formulation of leverage centrality. However, the inclusion and appropriate weighting of 2-, 3-, or n-hop neighbors in the leverage formulation would enable consideration of input signal from further upstream, as well as the propagation of signal further downstream in the network. Such an extension would provide further insight into the interdependence of nodes and may indeed be a more accurate model of a system such as the brain.

## Supporting Information

Text S1 (0.02 MB DOCX)

Figure S1 Jaccard index matrix comparing modularity results from 15 different QCUT runs. Note that runs 4, 6, and 7 have the lowest overall Jaccard index. The remaining runs have average Jaccard indices greater than 0.92. The diagonal is arbitrarily set to zero.
(0.33 MB TIF)

Figure S2 Three-dimensional scatter plots of degree, leverage, and eigenvector centrality. In all subjects, several groupings of nodes emerge.
(1.33 MB TIF)

Table S1 Summary of results from multiple realizations of QCUT run on a single complex brain network.
(0.03 MB DOC)
