# Supplementary material for: A New Measure of Centrality for Brain Networks
Source: PLoS One. 2010 Aug 16;5(8):e12200. doi: 10.1371/journal.pone.0012200 (PMC2922375; doi:10.1371/journal.pone.0012200)
Supplement: Text S1 — (0.02 MB DOCX) [file pone.0012200.s001.docx]

**Supporting Information**

*Reproducibility of QCUT Algorithm*

Modularity analyses necessarily use optimization algorithms to estimate community structure because it is thought to be an NP hard problem (Newman and Girvan, 2004). The QCUT method used here (Ruan and Zhang, 2008) is a top-down method that breaks the network from a complete graph into individual nodes resulting in a hierarchical structure. This method does not require a prior definition of the number of modules to be identified. QCUT is a spectral graph partitioning method that finds the second smallest eigenvector to identify the optimal division splitting the network into 2 or 3 parts. The method then optimizes the divisions by moving individual nodes and/or merging subdivisions. Each step is evaluated by calculating modularity (Q) to determine if the change is optimal. The resultant divisions are then recursively split into smaller subdivisions with optimization. The algorithm continues until Q can no longer be increased. The community structure that generates the highest Q is selected as the optimal structure. In the event that any particular subdivisions produce the same Q value, the division is randomly selected. Due to the optimization process and the potential need to randomly select a division, the community structure generated is different each time the algorithm is run. However, because the method starts with the full network, the first break will always be the same, that is the second smallest eigenvector is the same every time you start from the whole graph (unless there are multiple possible breaks that yield the same Q). The small changes that result from optimization are more susceptible to variability across realizations of the algorithm. Nevertheless, the major subdivisions will typically be fairly robust. Bottom-up methods start with all nodes isolated and begin adding edges to generate modular structure. The bottom-up methods can be sensitive to the random starting seed and can generate varied results based on initial conditions. This level of variability stems from the fact that one must start inserting edges between nodes at some point and the random selection of one edge can ultimately result in a different modular structure than an initial starting condition with another edge.

Using a single brain network we performed the QCUT modularity analysis 15 different times. This yielded 15 different modular structures each with a uniquely associated modularity value (Table S1). These data demonstrate that the number of modules identified ranged from 9-10 and that the Q ranged from 0.6719-0.6725. In fact, 9 of the 15 runs yielded the exact same Q. The modular structure from each run of the algorithm was then compared the modular structure of each other run using the Jaccard index. The Jaccard index matrix shown in Figure S1 demonstrated a very high level of agreement between the modular organizations across runs, with the mean Jaccard being 0.931. Even the worst agreement between any runs (7 and 13) yielded a Jaccard of 0.8603.

*Consistency of scatter plot spatial patterns*

Upon examining leverage, degree, and betweenness centrality on three-dimensional scatter plots, it became evident that there existed a particular group of nodes having lower leverage than would be expected based on a randomly connected graph. Similarly, a group of nodes having higher leverage emerged. Since leverage reflects local assortativeness of the network, local assortative and disassortative behavior resulted in this split of nodes having higher or lower leverage when compared to the synthetic network. Furthermore, when examining degree, leverage, and eigenvector centrality it became evident that those nodes with lower leverage than expected based on the synthetic network could be further subdivided. For the subject shown in the main text, two subgroups emerged from the lower leverage group, and the spatial patterns shown for this subject were qualitatively consistent with those seen in other subjects, and in some subjects a third subgroup was present. Three-dimensional plots for all subjects are shown in Figure S2.

References

Newman ME, Girvan M (2004). Finding and evaluating community structure in networks. *Phys Rev E Stat Nonlin Soft Matter Phys* 69, 026113.

Ruan J, Zhang W (2008). Identifying network communities with a high resolution. *Phys Rev E Stat Nonlin Soft Matter Phys* 77, 016104.
