# Supplementary figures and images for: A New Measure of Centrality for Brain Networks
Source: PLoS One. 2010 Aug 16;5(8):e12200. doi: 10.1371/journal.pone.0012200 (PMC2922375; doi:10.1371/journal.pone.0012200)

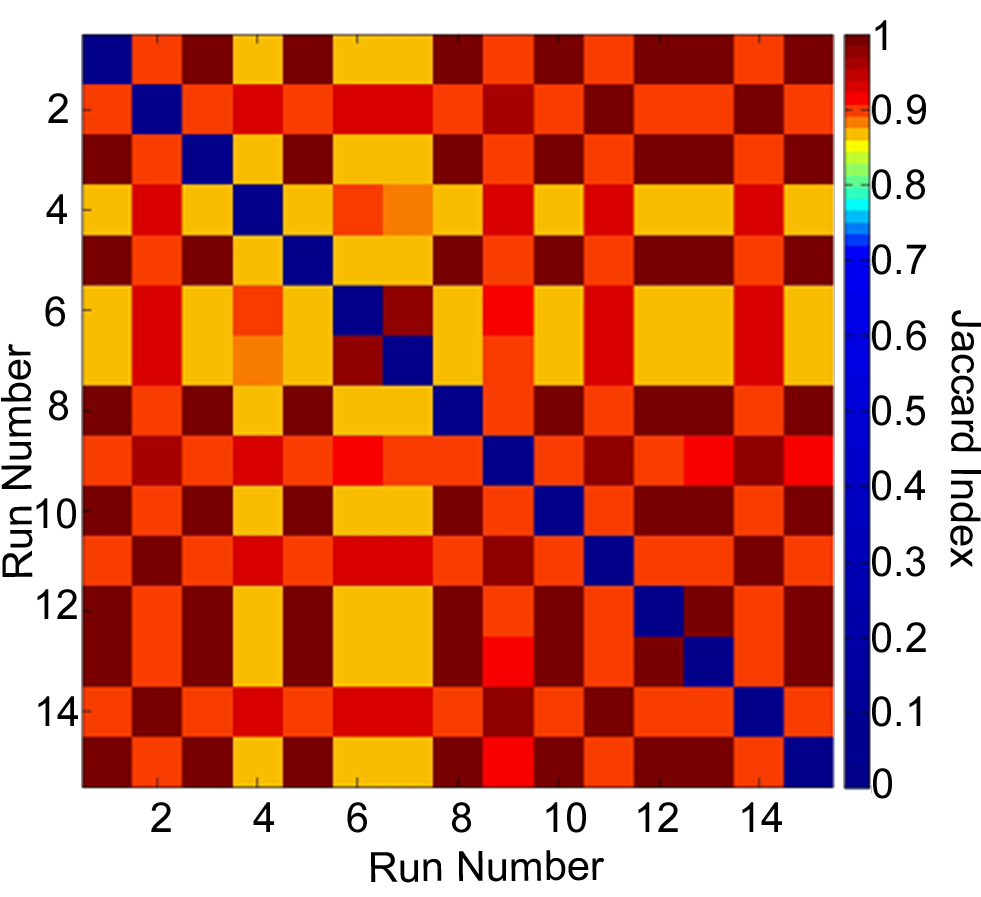

Supplement: Figure S1 — Jaccard index matrix comparing modularity results from 15 different QCUT runs. Note that runs 4, 6, and 7 have the lowest overall Jaccard index. The remaining runs have average Jaccard indices greater than 0.92. The diagonal is arbitrarily set to zero. (0.33 MB TIF) [file pone.0012200.s002.tif]

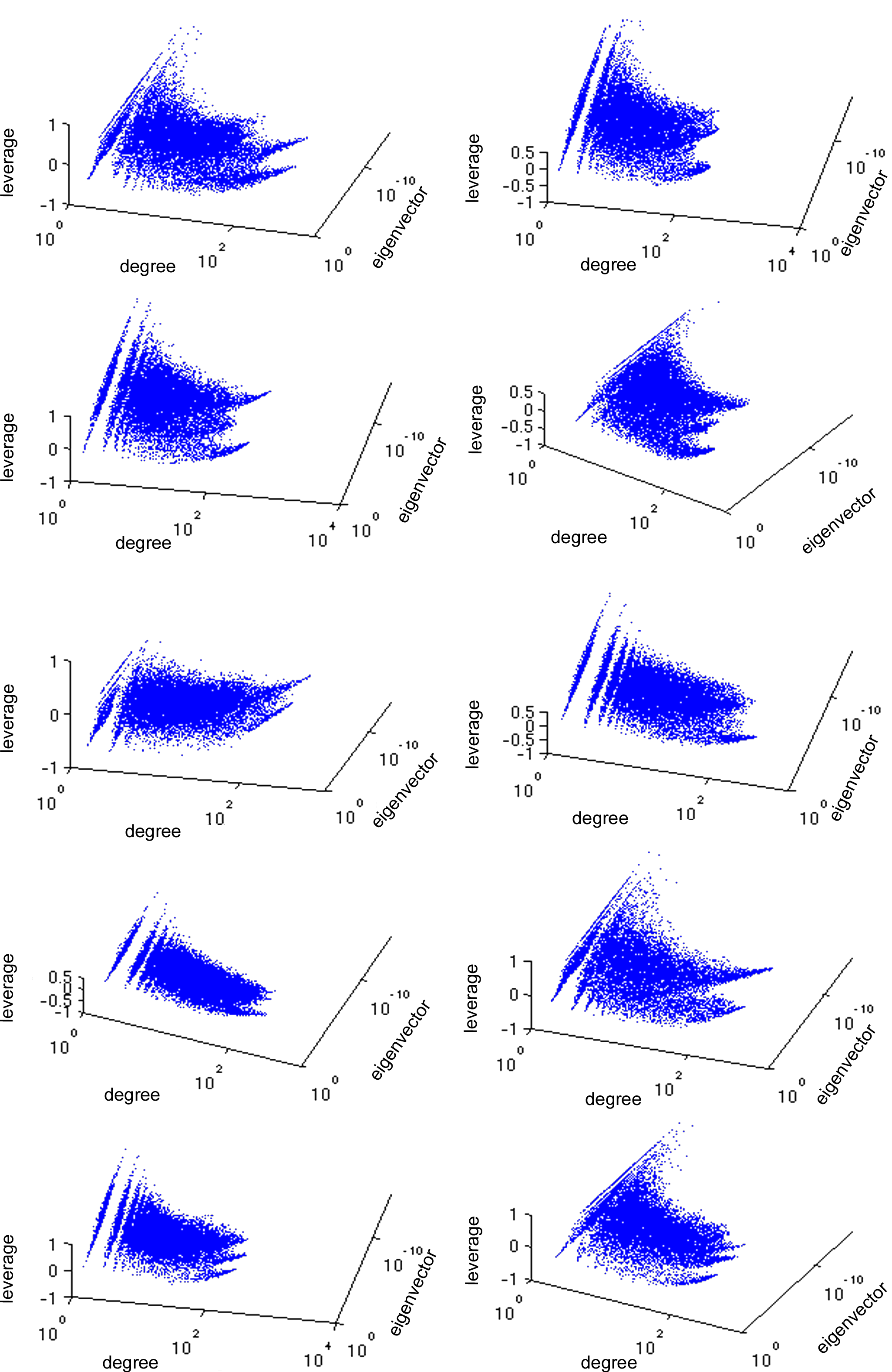

Supplement: Figure S2 — Three-dimensional scatter plots of degree, leverage, and eigenvector centrality. In all subjects, several groupings of nodes emerge. (1.33 MB TIF) [file pone.0012200.s003.tif]
