# Supplementary material for: A New Measure of Centrality for Brain Networks
Source: PLoS One. 2010 Aug 16;5(8):e12200. doi: 10.1371/journal.pone.0012200 (PMC2922375; doi:10.1371/journal.pone.0012200)
Supplement: Table S1 — Summary of results from multiple realizations of QCUT run on a single complex brain network. (0.03 MB DOC) [file pone.0012200.s004.doc]

| Run | Q | # of modules | Mean Jaccard |
| --- | --- | --- | --- |
| 1 | 0.6725 | 10 | 0.94451 |
| 2 | 0.6723 | 10 | 0.94451 |
| 3 | 0.6725 | 10 | 0.94513 |
| 4 | 0.6719 | 10 | 0.89697 |
| 5 | 0.6725 | 10 | 0.94546 |
| 6 | 0.6725 | 9 | 0.9025 |
| 7 | 0.6724 | 9 | 0.89673 |
| 8 | 0.6725 | 10 | 0.94513 |
| 9 | 0.6723 | 12 | 0.92579 |
| 10 | 0.6725 | 10 | 0.94562 |
| 11 | 0.6722 | 10 | 0.9302 |
| 12 | 0.6725 | 10 | 0.94595 |
| 13 | 0.6725 | 10 | 0.94124 |
| 14 | 0.6723 | 9 | 0.93092 |
| 15 | 0.6725 | 10 | 0.94183 |
